# Editorial: Photosynthesis in a changing global climate: A matter of scale, volume II

**DOI:** 10.3389/fpls.2023.1158816

**Published:** 2023-02-22

**Authors:** Marouane Baslam, Alvaro Sanz-Saez

**Affiliations:** ^1^ Laboratory of Biochemistry, Department of Applied Biological Chemistry, Faculty of Agriculture, University of Niigata, Niigata, Japan; ^2^ Centre d’Agrobiotechnologie et Bioingénierie, Unité de Recherche labellisée CNRST (Centre AgroBio-tech-URL-CNRST-05), Université Cadi Ayyad, Marrakech, Morocco; ^3^ Laboratory of Agro-Food, Biotechnologies and Valorization of Plant Bioresources (AGROBIOVAL), Department of Biology, Faculty of Science Semlalia, Cadi Ayyad University (UCA), Marrakesh, Morocco; ^4^ Department of Crop, Soil and Environmental Science, Auburn University, Auburn, AL, United States

**Keywords:** photosynthesis, climate change, crops, abiotic stress, productivity

Photosynthesis is the central process of all primary production in the biosphere. Photosynthesis in terrestrial plants absorbs nearly 123 Gt of atmospheric CO_2_ every year (C yr^-1^) and about half of the C fixed by leaves (c. 60 Gt C yr^-1^) is then returned to the atmosphere *via* autotrophic respiration (respiration by plant tissues) (Dusenge et al., 2019; Naseem et al., 2020; Walker et al., 2021). The CO_2_ assimilated by the photosynthetic apparatus is the basis of crop production and, therefore, all agricultural and human feed commodities. Over the last few years, numerous multidisciplinary research efforts have encouraged in-depth studies of the photosynthetic apparatus as a tool to increase the growth and production of plants under different environmental conditions. Photosynthesis is conditioned by the effect of environmental variables (water availability, temperature, [CO_2_], salinity, ozone, etc.) on light capture and utilization, the diffusion of CO_2_ from the atmosphere to the chloroplasts, the fixation of the CO_2_ by the enzyme Rubisco and the biochemical regeneration of the substrates of the Calvin Cycle (Long et al., 2015). Although it is generally accepted that stomatal closure is a target factor limiting photosynthetic activity under moderate stress conditions, when stress is more severe, it has been found that metabolic deterioration occurs. Variable phenotypic/genotypic responses to abiotic stress have been identified as targets to improve crop response to stress and as a tool to better understand the genetic mechanisms regulating such responses. Understanding the mechanisms and regulation of the photosynthetic processes together with the use of novel plant phenotyping technologies, specifically under abiotic stress conditions are crucial to identify novel targets/networks to improve photosynthetic efficiency. This understanding will ultimately lead toward designing and engineering crops that are adaptable to environmental changes in the realm of human population growth, globalization, and current and future climate change.

This Research Topic spans a range of studies at different levels of organization within photosynthetic research under a variety of environmental conditions geared towards improved yield and quality/functionality of plants grown under a changing global climate. The goals posed by four research groups using approaches from cell and molecular biology, genetics, biochemistry, structural biology, bioinformatics, eco-physiology, and engineering are focused on unraveling the complex network of genetic, biochemical, and metabolic interactions between the plant and the environment. An in-depth perspective article by Li et al. addresses the energy production (in)efficiency of plants and the energy cost of the process of respiration and maintenance under high-temperatures (HTs) conditions, and outlines strategies oriented toward engineering ‘smart crops’ with high energy utilization efficiency to reduce yield losses caused by HTs. Three research articles regarding this issue address topics related to the functional insights into the photosynthetic machinery and processes under abiotic stress conditions, as well as applied research aimed at mitigating the negative effects of climate change. These contributions concern both agronomic (e.g. rice and maize) and horticultural (e.g. citrus and orchid *Phalaenopsis*) -oriented materials research for a wide international audience. Jiang et al. provides insights into the molecular mechanism that contributes to drought tolerance of tetraploidy (vs. diploidy) in *Citrus wilsonii*. Through a combination of tools involving the use of microscopic observation, mRNAseq, phosphoproteomic, and hormonal quantification analyses, the authors reveal the synergistic regulation of photosynthesis, phosphorylation, and phytohormone accumulation in tetraploid tolerance to water limitations. Kang et al. describes short-term (substrate) and long-term (acclimation) effects of elevated CO_2_ (eCO_2_) on dynamic photosynthesis in two major C3 crop plants; rice and wheat, and the relative contribution of the acclimation and substrate effects of four different CO_2_ treatments on dynamic photosynthesis. In this study, neither an increased CO_2_ supply nor an acclimation to eCO_2_ imposes large influences on the photosynthetic induction rates, while an increased CO_2_ supply enhances photosynthetic C gain *via* improving steady-state net photosynthesis. These results provide a potential reason for the lower enhancement of yield in rice than in wheat under eCO_2_ conditions.

Addressing the abiotic stress responses in crassulacean acid metabolism (CAM), among the three main metabolic adaptations for CO_2_ fixation found in plants, one paper focuses on understanding the chilling sensitivity in the CAM orchid *Phalaenopsis*. Daems et al. describe the effects of chilling temperatures on photosynthetic performance in tropical CAM plants by consideration of different aspects of photosynthesis, including light reactions and carbon fixation. Understanding the physiological responses of CAM plants to chilling will help facilitate the design and development of breeding strategies as well as enhance efforts to increase sustainability in the horticultural sector.

This Research Topic presents examples of cross-disciplinary research that have utilized concepts from multi-’omics’, experimental biology, and eco-physiology to illustrate how photosynthesis research can help to mitigate abiotic stresses and enhance food safety. We hope these exciting new works serve to inform, answer some long-standing questions (while raising many new ones), and inspire researchers in key areas of plant abiotic stress, climate change, and molecular biology. Please read and enjoy.

We encourage authors to continue to submit their best work in plant abiotic stress biology to The Frontiers in Plant Sciences.

## Author contributions

All authors critically read and edited the manuscript. All authors contributed to the article and approved the submitted version.
